# What Counts for the Old and Oldest Old?—An Analysis of Patient Criteria for Choosing a Dentist—Part II: Personal Characteristics and Soft Skills

**DOI:** 10.3390/ijerph19148621

**Published:** 2022-07-15

**Authors:** Ina Nitschke, Thomas Ulbrich, Annett Schrock, Werner Hopfenmüller, Julia Jockusch

**Affiliations:** 1 Gerodontology Section, Department of Prosthodontics and Materials Science, University of Leipzig, Liebigstraße 12, 04103 Leipzig, Germany; ina.nitschke@medizin.uni-leipzig.de (I.N.); thomas.ulbrich@medizin.uni-leipzig.de (T.U.); annett.schrock@medizin.uni-leipzig.de (A.S.); 2 Clinic of General, Special Care and Geriatric Dentistry, Center of Dental Medicine, University of Zurich, Plattenstrasse 11, CH-8032 Zurich, Switzerland; 3 Institute of Biometry and Clinical Epidemiology, Charité—Universitätsmedizin Berlin, Corporate Member of Freie Universität Berlin, Humboldt-Universität zu Berlin, and Berlin Institute of Health, 10117 Berlin, Germany; werner.hopfenmueller@charite.de; 4 University Research Priority Program “Dynamics of Healthy Aging”, University of Zurich, CH-8050 Zurich, Switzerland

**Keywords:** ideal dentist, patients’ perceptions, influencing factors, telephone survey, soft skills, psycho-social competence

## Abstract

Soft skills include communication skills and personality traits that are important when choosing a dentist, but other factors within the dental office also seem to be important for patients. The aim of this study is to evaluate factors that are important to people in a dentist as well as characteristics of the ideal dentist and to evaluate possible age-, gender-, and residence of living specific differences. A telephone survey with participants aged 35 years or older (ag—age group: ag 1: 35–50 years, ag 2: 70–84 years, ag 3: >85 years) in three German cities was conducted. Data were analyzed with respect to gender and age. Most of the participants (*n* = 298, 64.2%), regardless of their own gender, age, or place of residence did not care about the gender of the dentist. In general, the price of the treatment does not play a role in choosing the ideal dentist. Women differ significantly from men in their choice of dentist (ANOVA *p* < 0.001 (preference of non-smoker), ANOVA *p* < 0.001 (preference, that the dentist does not smell of smoke, importance of appearance (ANOVA *p* < 0.001) and psycho-social skills, etc.). As age increases, professional experience and psycho-social competencies are rated as important. With the increase in age, the mean value of the desired years of professional experience increases without significant differences between age groups. The importance of advanced training (ANOVA *p* < 0.001; Bonferoni correction: significant difference between ag 1 and ag 2 *p* < 0.001, and ag 1 and ag 3 *p* < 0.001) decreases with age. Especially for participants aged 70 to 84 years, a relationship of trust is important. Between the places of residence, statistical differences for almost all surveyed items were found (e.g., importance that the dentist speaks the patients’ native language ANOVA *p* < 0.001, Bonferoni correction: significant difference between Berlin and Leipzig, Berlin and Mainz, and Leipzig and Mainz (each *p* < 0.001), dentist has a specialization ANOVA *p* < 0.001, Bonferoni correction: significant difference between Berlin and Leipzig and Berlin and Mainz (each *p* < 0.001), etc.). Dentists should be trained to develop psycho-social skills to meet the special demands of the increasing older population.

## 1. Introduction

As early as 1959, the first scientists began to think about the ideal doctor [1], and this topic has been occupying the medical profession ever since [2,3,4,5]. To maintain or improve the quality of dental treatment, it is important that the training of dentists is constantly adapted to the current state of knowledge. However, patient-centered care needs to focus on more aspects than on clinical treatment outcomes and patient safety [6]. Patients will benefit from patient-centered healthcare [7] which positively influences their physical and psychological outcomes [8]. On the other hand, in addition to professional aspects, so-called soft skills, such as communication skills [3] or psycho-social skills [4] of the dentist, his or her personality traits, as well as factors within the dental office [9] seem to be important for patients. Additionally, the direct involvment of patients in the decision-making process should be emphasized [10] as preferred by most patients [5].

It can be assumed that dentists and patients have a different image of an ideal dentist. Various studies have shown that patients rated the physical characteristics of the dental office premises as well as the interaction with the staff as the priority issues [9]. Lahti et al. were able to show that both parties agree on the characteristics of an ideal dentist only regarding the characteristic’s “communicativeness” and “informativeness” [2,11]. When patients were asked what their ideal dentist looked like, characteristics such as the professional competence [5,12,13], treatment quality [13], the ability “to put the patient at ease”, patience and respect [13,14], participation of the patient in the decision-making and treatment process, as well as explanation of the treatment [5] and friendliness were mostly associated with the ideal dentist [12].

The influence of social media appears to be less decisive for the choice of a surgeon than a personal recommendation [13,15] and the quality of the information that is provided at the time of the consultation [15].

Empathy seems to play a key role in the ideal dentist–patient relationship [14,16]. It may positivley influence the patients’ adherence to therapy as well as the patients’ satisfaction. Furthermore, empathetic communication may reduce dental anxiety [16].

With the increase in the importance of age- and gender-specific influences due to the current demographic change and the focus in the field of gender medicine, it is also becoming important for dentistry to know what differences exist in the requirements for an ideal dentist. To overcome the barriers in the provision of dental services and increase the utilization—especially for the old and very old—is becoming more important [17].

Increasing the utilization of dental services can serve to start or maintain preventive measures. Patient-centered dentistry should therefore consider all factors that encourage patients to use dental services. Seniors, in particular, are less likely to use dental services as they age or as they become more frail or cognitively ill [17,18,19]. Knowing what characterizes an ideal dentist–patient relationship and what factors influence utilization is therefore immensely important for dentists—regardless of the economic considerations involved in acquiring patients.

The aim of the study is to evaluate factors that are important to patients in a dentist as well as characteristics of the ideal dentist and to evaluate possible age- and gender-specific differences.

## 2. Materials and Methods

The analyzed data are part of a telephone survey in three German cities (Berlin (capital of Germany), Leipzig (East Germany), and Mainz (West Germany) on important aspects and characteristics of an ideal dentist.

Participants aged 35 years and older (age group (ag): ag 1: 35–50 years, ag 2: 70–84 years, ag 3: >85 years) were included for participation in the survey; an upper age limit was not defined. No further inclusion and exclusion criteria for participation were applied except that participants had to be able to understand and answer questions in German. The participants were selected by a random sample (sample size *n* = 1400 for each city) of the responsible municipal personal registration offices. All participants were interviewed by telephone over a period of one year (2012–2013). The time required per respondent was 10–15 min.

The interviews were conducted with three interviewers. All interviewers were trained on the content of the interview and how to conduct a telephone interview. To assure quality of the interviews, before the study, all interviewers conducted pretest interviews without knowing. The pretest interviews were then discussed with the study director before the start of the interviews for this study.

For the first part of the analysis (Important aspects of an “ideal dentist” and Importance of human interactions as criteria for the choice of a dentist), 5-point Likert scales were used (criteria: very important, important, partly/partly, unimportant, very unimportant). On the other hand, close-ended questions were asked about: (a) the preference regarding the gender of the ideal dentist (categories: female, male, gender is irrelevant), (b) the existence of age preferences (categories: yes, no) and the indication of the preferred age of the dentist in years, and (c) the desired professional experience of the dentist in years.

For the second part of the analysis (Characteristics of an “ideal dentist”), open-ended questions were asked about the desired characteristics of the dentist. Respondents could name up to three characteristics in order of personal importance. These were assigned to categories for the evaluation (categories: psycho-social competencies/personality characteristics; professional competence, working methods and quality; price for dental treatment, others) and evaluated separately according to gender and age groups. First to third mentions per age group were cumulated according to the categories. Accordingly, multiple answers within one and the same category were possible for one participant.

The statistical analysis was carried out with SPSS (version 27.0, IBM, Chicago, IL, USA) [20]. For the evaluation, three age groups (ag 1: 35–50 years, ag 2: 70–84 years and ag 3: >85 years) were defined, gender-specific differences (male, female), and different residences of living (Berlin, Leipzig, Mainz) were evaluated. The statistical evaluation was carried out descriptively for absolute frequencies. Analysis of variance (ANOVA) with Bonferoni correction was employed to compare the different groups. The significance level was set at *p* < 0.05.

The study was conducted according to the guidelines of the Declaration of Helsinki and approved by the competent Ethics Committee of the University of Leipzig (study number: 135-11-ff-18042011).

## 3. Results

Randomly selected for this survey were 1400 addresses per city (total *n* = 4200). A total of 2889 telephone numbers (72.2%) were identified, of which a total of 1746 potential participants (60.4%) were being contacted. It was not possible to identify the remaining telephone numbers. From all contacted participants, 1280 participants (73.3%) rejected to participate in the telephone survey (reasons: lack of time, lack of interest, difficulties in communicating by phone).

A total of 466 participants (male *n* = 187, 40.1%; female *n* = 279, 59.9%) have been included in the analysis. They belong almost equally to one of the three age groups (ag 1: *n* = 152, 32.6% (mean age: 43.3 ± 4.6 years); ag 2: *n* = 155, 33.3% (mean age: 77.3 ± 4.3 years); ag 3: *n* = 159, 34.1% (mean age: 88.9 ± 2.8 years).

Data on patients’ perceptions regarding awareness and selection criteria, infrastructure, and dental office equipment can be found in part I of the data analysis [21].

### 3.1. Important Aspects of an “Ideal Dentist”

It was important or very important to most participants irrespective of age, gender, or place of residence that the dentist and his team speak the patient’s mother tongue. Especially, as age increases, it becomes more important that the dentist speaks the patient’s mother tongue (ANOVA *p* = 0.016; Bonferoni correction: significant difference between ag 1 and ag 2 *p* = 0.006). Between the places of residence, a significant difference occurred for this item (ANOVA *p* < 0.001; Bonferoni correction: significant difference between Berlin and Leipzig *p* < 0.001, Berlin and Mainz *p* < 0.001, and Mainz and Leipzig *p* < 0.001). A dentist who speaks the patient’s mother tongue is almost twice as important for participants living in Berlin or Mainz than those living in Leipzig (Table 1).

Whether the dentist should: (a) be a non-smoker, and (b) not smell of smoke is significantly more important to women than to men (ANOVA *p* < 0.001 (question: non-smoker), ANOVA *p* < 0.001 (question: do not smell of smoke)). Significant differences also occurred between the place of residence (ANOVA each item *p* < 0.001; Bonferoni correction: significant difference between Berlin and Leipzig *p* < 0.001, and Berlin and Mainz *p* < 0.001 (question: non-smoker), Berlin and Leipzig *p* < 0.001, and Leipzig and Mainz *p* < 0.001 (question: do not smell of smoke)) (Table 1).

Most of the participants (*n* = 298, 64.2%), regardless of their own gender, age, or place of residence did not care about the gender of the dentist (Table 1).

Regarding the desired professional experience of the dentist, no differences were found with regard to age, gender, or place of residence. With the increase in age, the mean value of the desired years of professional experience increased without significant differences between age groups. Significant differences occurred between the places of residence (ANOVA *p* = 0.011; Bonferoni correction: significant difference between Berlin and Leipzig *p* = 0.009) (Table 1).

The appearance of the dentist (ANOVA *p* < 0.001) and the patience of the dentist during treatment (ANOVA *p* = 0.003) are more important to women than to men. Significant differences for the item “patience during treatment” were also observed between the places of residence (ANOVA *p* < 0.001; Bonferoni correction: significant difference between Berlin and Leipzig *p* < 0.001, and Berlin and Mainz *p* < 0.001), and between the age groups (ANOVA *p* = 0.031; Bonferoni correction: significant difference between ag 1 and ag 3 *p* = 0.018) (Table 1).

Whether the dentist has advanced training is less important as age increases (ANOVA *p* < 0.001; Bonferoni correction: significant difference between ag 1 and ag 2 *p* < 0.001, and ag 1 and ag 3 *p* < 0.001). A relationship of trust with the dentist is most important to ag 2 participants (ANOVA *p* = 0.023; Bonferoni correction: significant difference between ag 1 and ag 2 *p* = 0.04, and ag 1 and ag 3 *p* = 0.024) (Table 1).

### 3.2. Importance of Human Interactions as Criteria for the Choice of a Dentist

For male participants, a welcoming reception at the front desk is more important than for female participants (ANOVA *p* > 0.001). In contrast, female participants consider friendly interaction between the dentist and his team more important than males (ANOVA *p* = 0.002). Significant differences also occurred between the places of residence (ANOVA each *p* > 0.001; Bonferoni correction: significant difference between Berlin and Leipzig *p* < 0.001, Berlin and Mainz *p* = 0.003, and Leipzig and Mainz *p* = 0.002 (item welcoming reception); Berlin and Leipzig *p* < 0.001, and Leipzig and Mainz *p* < 0.001 (item friendly interaction)) but not between age groups (Table 1).

### 3.3. Characteristics of an “Ideal Dentist”

A total of 457 (98.1%) of the participants named at least one characteristic, 408 participants (87.6%) named two characteristics, and 276 participants (59.2%) named three characteristics that they would like to see in their dentist.

Regardless of age or gender, the majority of respondents (*n* = 457) named psycho-social skills (*n* = 257, 56.2%) as the desired characteristic of their dentist in the first position of the mentions, followed by professional competence, working methods and quality (*n* = 181, 39.6%), other (e.g., proximity of the practice etc., *n* = 11, 2.4%) and the factor “price” (*n* = 8, 1.8%). Psycho-social skills (second naming: *n* = 242, 59.3%; third naming: *n* = 159, 57.6%) and professional skills, working methods and quality (second naming: *n* = 122, 2.4%; third naming: *n* = 159, 57.6%) were also in the lead when it came to the second and third naming of a desired characteristic. Second naming: *n* = 122, 29.9%; third naming: *n* = 93, 33.7%) before price (second naming: *n* = 27, 6.6%; third naming: *n* = 16, 5.8%) or other naming (second naming: *n* = 17, 4.2%; third naming: *n* = 8, 2.9%).

**Figure 1:**
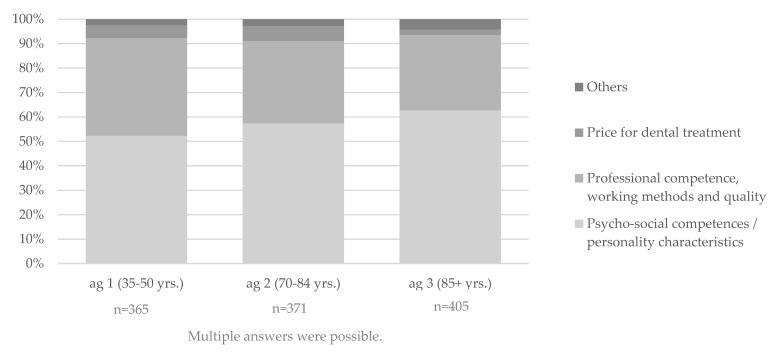
Naming of characteristics of an ideal dentist graded according to importance (first mention to third mention) and separated by age group (ag). First to third mentions per age group were cumulated according to the categories. Accordingly, multiple answers within one and the same category were possible for one participant.

With the increase in age, there is a tendency towards an increase in the importance of psycho-social competencies. At the same time, the importance of professional competence decreases with the increase in age (first mention). Overall, price plays a subordinate role. In ag 3, it is least important compared to ag 1 and ag 2 (Figure 1).

**Figure 2:**
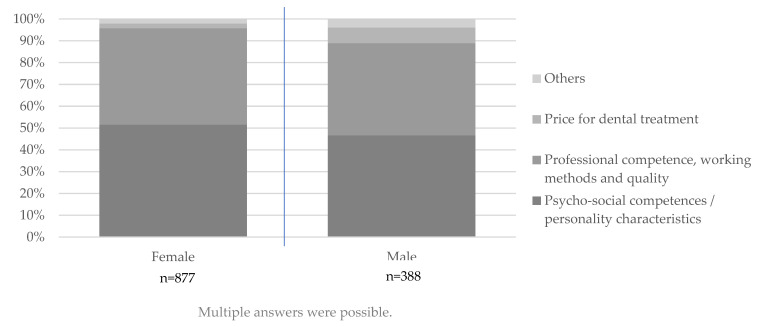
Naming of characteristics of an ideal dentist graded according to importance (first mention to third mention) and separated by gender. First to third mentions per age group were cumulated according to the categories. Accordingly, multiple answers within one and the same category were possible for one participant.

Psycho-social competencies are clearly more important for women than for men, while men state professional competence, working methods and quality as a more desirable characteristic of the dentist. Price also plays a subordinate role for the sexes, but men name it more frequently as a criterion than women. Overall, women and men differ significantly in the naming of the desired characteristics for all three naming’s (Pearson Chi Square test: first naming *p* < 0.01, second naming *p* = 0.028, third naming *p* = 0.034) (Figure 2).

## 4. Discussion

### 4.1. Study Limitations

The overall response rate was around two-thirds in this study. For a telephone survey, this is an acceptable rate. However, the literature reveals that due to changes in culture, marketing and the entire field of telecommunications, response rates for telephone surveys are declining [22]. As a result, the conclusions drawn in this study should be carefully interpreted.

Additionally, a bias cannot be ruled out since it is possible that only people who are interested in a dentist or a dental visit may have taken part in the survey. Therefore, maybe not all aspects of an ideal dentist have been evaluated.

A difficulty lies within the survey instrument itself. It is possible that questions were not understood as well over the telephone. Moreover, a lack of visual support during answer selection might have influenced the answers. Often in telephone surveys, predominantly first- or last-order answer choices are selected. Furthermore, expected social desirability cannot be excluded when analyzing the answers given by the participants.

### 4.2. Comparison with Other Studies

Irrespective of age, gender, or place of residence, the gender of the dentist does not play a role in this study. Meanwhile, 45% of respondents in a study by Fennema et al. indicated preferences regarding the gender of the physician. The majority preferred their own gender when choosing a physician [23].

As already shown by Lahti et al., the ideal dentist is characterized by several features for patients. In addition to psycho-social skills (in Lahti et al. “mutual communication”, “fair support” and “blaming”) [2,3,11], the present study showed that participants attach importance to personal appearance. For example, appearance was just as important to women as the fact that the dentist is a non-smoker or does not smell of smoke.

In the present study, it was particularly important for women that the dentist had psycho-social skills as well as patience during treatment. This is consistent with the observation of Van Groenestijn et al. that although women go to the dentist more regularly than men, as a group they refer more often to reassurance than to professional competence [12]. In addition, Lamprecht et al. reported, that psycho-social skills of the dentist are most important to the patient when choosing a dentist [4].

A psycho-social ability of enormous importance seems to be the empathy of the dentist [16]. It has been shown that empathy plays a key role in retaining patients in therapy and increasing their satisfaction. At the same time, the method of applied empathy can reduce dental anxiety. Jones et al. describe that empathy plays a crucial role in differentiating between informative and emotional communication. Dentists should therefore be taught this concept [16].

In the literature, the ideal physician is also described as self-aware, empathetic, humane, personable, open, respectful, and thorough [14].

While the literature reports professional competence as one of the most frequently mentioned positive characteristics of the ideal dentist [5,12,13], this could not be confirmed in the present study. Only men attached more importance to the quality of work than, for example, to the psycho-social characteristics of the dentist. However, with the increase in age, professional experience became more important, and the importance of advanced training and professional competence decreased.

Interpreting the negative characteristic roughness of the dentist as an expression of a lack of psycho-social competence, it also seems to be important in the study by Van Groenestijn et al. that psycho-social competencies are available or desired [12]. The present study also showed that the importance of the dentist’s psycho-social skills increases with the increase in the age of the participants.

Meanwhile, dental cost played a minor role in the present study for all but predominantly younger men, but cost was among the three most frequently mentioned negative characteristics in Van Groenestijn et al. [12]. Van Groenestijn et al. assume that many of the characteristics attributed to the ideal dentist are the result of experience and working conditions imposed externally on the dentist (e.g., third-party payment, etc.). However, it seems that, on the one hand, the way people perceive the dentist’s task and, on the other hand, the attitude towards dental care in general have an influence on the image of the ideal dentist [12].

Future studies should also consider other factors on the participants’ side to paint a more complete picture of an ideal dentist. In addition to previous experience with dentists [3], variables such as the participant’s own utilization behavior (control-oriented versus complaint-oriented) or living situation (living at home, needing outpatient care, living in a care facility) should also be considered regarding the aging of the society.

## 5. Conclusions

In general, neither the gender of the dentist nor the price of the treatment plays a role for the selection of a dentist. Women differ from men in their choice of dentist. They prefer a non-smoker as a dentist, dentists who do not smell of smoke, and attach importance to appearance. Patience during treatment and psycho-social skills are more important to women, while quality of work is more important to men. As age increases, it becomes more important that the dentist speaks one’s mother tongue. Furthermore, professional experience is important, while the importance of advanced training and professional competence decreases. Especially for participants aged 70 to 84 years, a relationship of trust is important. Overall, the psycho-social competencies of the dentist are considered important as the age of the participants increases. Therefore, dentists should be trained to develop psycho-social skills to meet the demands of the increasing older population.

## Figures and Tables

**Table 1:** Important aspects of an ideal dentist separated by gender (female *n* = 279, male *n* = 187), age group (ag 1 *n* = 152, ag 2 *n* = 155, ag 3 *n* = 159), and place of residence (Berlin *n* = 152, Leipzig *n* = 150, Mainz *n* = 164). (Total (all participants) *n* = 466; n/%—number/percent, ag—age groups; bold values in column *p* indicate statistical significance with a significance level of *p* < 0.05, *p* = ANOVA with Bonferoni correction for age group and place of residence/without Bonferoni post hoc test for sex. * Indicates the differences between two cities or age groups.

	Total	Sex			Age Group				Residence of Living				R^2^
	All [n/%]	Female [n/%]	Male [n/%]	[p]	ag 1 35–50 yrs. [n/%]	ag 2 70–84 yrs. [n/%]	ag 3 85 + yrs. [n/%]	[p]	Berlin (B) [n/%]	Leipzig (L) [n/%]	Mainz (M) [n/%]	[p]	
**How important is it that the dentist…**													
… and his team are able to speak the patients’ native language?													
	*n* = 462	*n* = 275	*n* = 187	0.175	*n* = 150	*n* = 154	*n* = 158	ANOVA **0.016** Bonferoni ag 1 * ag 2 **0.006**	*n* = 151	*n* = 149	*n* = 162	ANOVA **<0.001** Bonferoni B * L **< 0.001** B * M **< 0.001** M * L **< 0.001**	0.217
Very unimportant	2/0.4	1/0.4	1/0.5		1/0.7	0/0	1/0.6		2/1.3	0/0	0/0		
Unimportant	25/5.4	14/5.1	11/5.9		16/10.7	4/2.6	5/3.2		1/0.7	18/12.1	6/3.7		
Partly/partly	69/15.0	33/11.8	36/19.3		28/18.7	21/13.6	20/12.7		4/2.6	48/32.2	17/10.5		
Important	279/60.4	174/62.4	105/56.1		78/52.0	96/62.3	105/66.5		86/57.0	70/47.0	123/75.9		
Very important	87/18.8	53/19.0	34/18.2		27/18.0	33/21.4	27/17.1		58/38.4	13/8.7	16/9.9		
… is non-smoker?													
	*n* = 463	*n* = 276	*n* = 187	**<0.001**	*n* = 151	*n* = 154	*n* = 158	0.856	*n* = 152	*n* = 149	*n* = 162	ANOVA **<0.001** Bonferoni B * L **< 0.001** B * M **< 0.001**	0.126
Very unimportant	39/8.3	20/7.2	19/10.2		14/9.3	13/8.4	12/7.6		34/22.4	2/1.3	3/1.9		
Unimportant	149/32.2	78/28.3	71/38.0		53/35.1	42/27.3	54/34.2		48/31.6	60/40.3	41/25.3		
Partly/partly	98/21.2	51/18.5	47/25.1		32/21.2	38/24.7	28/17.7		30/19.7	37/24.8	31/19.1		
Important	130/28.1	92/33.3	38/20.3		34/22.5	46/29.9	50/31.6		29/19.1	27/18.1	74/45.7		
Very important	47/10.2	35/12.7	12/6.4		18/11.9	15/9.7	14/8.9		11/7.2	23/15.4	13/8.0		
… does not smell of smoke?													
	*n* = 463	*n* = 276	*n* = 187	**<0.001**	*n* = 151	*n* = 154	*n* = 158	0.638	*n* = 152	*n* = 149	*n* = 162	ANOVA **<0.001** Bonferoni B * L **< 0.001** L * M **< 0.001**	0.195
Very unimportant	9/1.9	4/1.4	5/2.7		3/2.0	4/2.6	2/1.3		4/2.6	3/2.0	2/1.2		
Unimportant	57/12.3	19/6.9	38/20.3		16/10.6	19/12.3	22/13.9		7/4.6	39/26.2	11/6.8		
Partly/partly	47/10.2	18/6.5	29/15.5		19/12.6	14/9.1	14/8.9		12/7.9	30/20.1	5/3.1		
Important	224/48.4	147/53.3	77/41.2		70/46.4	78/50.6	76/48.1		61/40.1	45/30.2	118/72.8		
Very important	126/27.2	88/31.9	38/20.3		43/28.5	39/25.3	44/27.8		68/44.7	32/21.5	26/16.0		
… has a well-groomed appearance?													
	*n* = 465	*n* = 278	*n* = 187	**<0.001**	*n* = 152	*n* = 155	*n* = 158	ANOVA **0.04** Bonferoni ag 1 * ag 3 0.084	*n* = 152	*n* = 149	*n* = 164	ANOVA **<0.001** Bonferoni B * L **< 0.001** B * M **< 0.001** L * M **< 0.001**	0.296
Very unimportant	0/0	0/0	0/0		0/0	0/0	0/0		0/0	0/0	0/0		
Unimportant	7/1.5	2/0.7	5/2.7		2/1.3	2/1.3	3/1.9		1/0.7	2/1.3	4/2.4		
Partly/partly	47/10.1	8/2.9	39/20.9		13/8.6	13/8.4	21/13.3		0/0	44/29.5	3/1.8		
Important	295/63.4	192/69.1	103/55.1		91/59.9	104/67.1	100/63.3		83/54.6	79/52.3	134/81.7		
Very important	116/25.0	76/27.3	40/21.4		46/30.3	36/23.2	34/21.5		68/44.7	25/16.8	23/14.0		
… has a professional specialization?													
	*n* = 464	*n* = 278	*n* = 186	0.157	*n* = 151	*n* = 155	*n* = 158	0.123	*n* = 152	*n* = 149	*n* = 163	ANOVA **<0.001** Bonferoni B * L **< 0.001** B * M **< 0.001**	0.083
Very unimportant	8/4.1	9/3.2	8/4.3		5/3.3	6/3.9	6/3.8		16/10.5	0/0	1/0.6		
Unimportant	94/20.3	53/19.1	41/22.0		24/15.9	38/24.5	32/20.3		41/27.0	24/16.1	29/17.8		
Partly/partly	205/44.2	121/43.5	84/45.2		66/43.7	64/41.3	75/47.5		57/37.5	82/55.0	66/40.5		
Important	127/27.4	82/29.5	45/24.2		44/29.1	42/27.1	41/25.9		30/19.7	33/22.1	64/39.3		
Very important	21/4.5	13/4.7	8/4.3		12/7.9	5/3.2	4/2.5		8/5.3	10/6.7	3/1.8		
… participates in continuing education?													
	*n* = 461	*n* = 277	*n* = 184	0.924	*n* = 150	*n* = 154	*n* = 157	ANOVA **<0.001** Bonferoni ag 1 * ag 2 **<0.001** ag 1 * ag 3**<0.001**	*n* = 152	*n* = 149	*n* = 160	ANOVA **<0.001** Bonferoni B * M **0.019** L * M **< 0.001**	0.164
Very unimportant	4/0.7	3/1.1	1/0.5		1/0.7	2/1.3	1/0.6		3/2.0	1/0.7	0/0		
Unimportant	28/6.1	16/5.8	12/6.5		3/2.0	13/8.4	12/7.6		17/11.2	8/5.4	3/1.9		
Partly/partly	92/20.0	49/17.7	43/23.4		16/10.7	34/22.1	42/26.8		21/13.8	63/42.3	8/5.0		
Important	269/58.4	172/62.1	97/52.7		92/61.3	89/57.8	88/56.1		81/53.3	54/36.2	134/83.8		
Very important	68/14.8	37/13.4	31/16.8		38/25.3	16/10.4	14/8.9		30/19.7	23/15.4	15/9.4		
… maintains a relationship of trust with you?													
	*n* = 463	*n* = 276	*n* = 187	0.058	*n* = 152	*n* = 155	*n* = 159	ANOVA **0.023** Bonferoni ag 1 * ag 2 **0.040** ag 1 * ag 3 **0.024**	*n* = 152	*n* = 149	*n* = 162	ANOVA **<0.001** Bonferoni B * M **< 0.001**	0.097
Very unimportant	1/0.2	1/0.4	0/0		1/0.7	0/0	0/0		1/0.7	0/0	0/0		
Unimportant	1/0.2	1/0.4	0/0		1/0.7	0/0	0/0		0/0	0/0	1/0.6		
Partly/partly	9/2.0	2/0.7	7/3.7		4/2.6	0/0	5/3.2		5/3.3	3/2.0	1/0.6		
Important	222/47.9	128/46.4	94/50.3		53/34.9	89/58.2	80/50.6		48/31.6	71/47.7	103/63.6		
Very important	230/49.7	144/52.2	86/46.0		93/61.2	64/41.8	73/46.2		98/64.5	75/50.3	57/35.2		
… starts his treatment on time?													
	*n* = 463	*n* = 276	*n* = 187	0.561	*n* = 151	*n* = 155	*n* = 157	0.367	*n* = 151	*n* = 148	*n* = 164	ANOVA **0.037** Bonferoni B * M **0.035**	0.063
Very unimportant	2/0.4	2/0.7	0/0		0/0	0/0	2/1.3		2/1.3	0/0	0/0		
Unimportant	26/5.6	18/6.5	8/4.3		7/4.6	12/7.7	7/4.5		10/6.6	0/0	16/9.8		
Partly/partly	156/33.7	89/32.2	67/35.8		48/31.8	54/34.8	54/34.4		41/27.2	67/45.3	48/29.3		
Important	232/50.1	142/51.4	90/48.1		78/51.7	77/49.7	77/49.0		67/44.4	71/48.0	94/57.3		
Very important	47/10.2	25/9.1	22/11.8		18/11.9	12/7.7	17/10.8		31/20.5	10/6.8	6/3.7		
… has enough time for his treatment?													
	*n* = 464	*n* = 277	*n* = 187	0.407	*n* = 152	*n* = 155	*n* = 157	ANOVA 0.068 Bonferoni ag 1 * ag 3 0.017	*n* = 152	*n* = 148	*n* = 164	0.150	0.069
Very unimportant	0/	0/0	0/0		0/0	0/0	0/0		0/0	0/0	0/0		
Unimportant	4/0.8	3/1.1	1/0.5		2/1.3	2/1.3	0/0		4/2.6	0/0	0/0		
Partly/partly	25/5.4	10/3.6	15/8.0		6/3.9	10/6.5	9/5.7		10/6.6	11/7.4	4/2.4		
Important	301/64.9	184/66.4	117/62.6		88/57.9	98/63.2	115/73.2		78/51.3	93/62.8	130/79.3		
Very important	134/28.9	80/28.9	54/28.9		56/36.8	45/29.0	33/21.0		60/39.5	44/29.7	30/18.3		
… has patience during treatment?													
	*n* = 464	*n* = 277	*n* = 187	**0.003**	*n* = 152	*n* = 155	*n* = 157	ANOVA **0.031** Bonferoni ag 1 * ag 3 **0.018**	*n* = 152	*n* = 148	*n* = 164	ANOVA **<0.001** Bonferoni B * L **< 0.001** B * M **< 0.001**	0.119
Very unimportant	0/0	0/0	0/0		0/0	0/0	0/0		0/0	0/0	0/0		
Unimportant	4/0.9	2/0.7	2/1.1		3/2.0	1/0.6	0/0		3/2.0	1/0.7	0/0		
Partly/partly	20/4.3	7/2.5	13/7.0		3/2.0	10/6.5	7/4.5		5/3.3	14/9.5	1/0.6		
Important	285/61.4	166/59.9	119/63.6		82/53.9	94/60.6	109/69.4		67/44.1	89/60.1	129/78.7		
Very important	155/33.4	102/36.8	53/28.3		64/42.1	50/32.3	41/26.1		77/50.7	44/29.7	34/20.7		
**Is there…**													
… a gender preference when choosing a dentist?													
	*n* = 464	*n* = 279	*n* = 185	0.974	*n* = 151	*n* = 154	*n* = 159	0.412	*n* = 151	*n* = 150	*n* = 163	0.128	0.031
Female dentist	56/12.1	28/10.0	28/15.1		22/14.6	17/11.0	17/10.7		21/13.9	23/15.3	12/7.4		
Male dentist	110/23.7	71/25.4	39/21.1		31/20.5	45/29.2	34/21.4		32/21.2	41/27.3	37/22.7		
Irrelevant	298/64.2	180/64.5	118/63.8		98/64.9	92/59.4	108/67.9		98/64.9	86/57.3	114/69.9		
… an age preference when choosing a dentist? (preferred age in years: Median (Range))													
	*n* = 463	*n* = 278	*n* = 185	0.506	*n* = 152	*n* = 155	*n* = 159	0.323	*n* = 150	*n* = 150	*n* = 163	ANOVA **0.011** Bonferoni B * L **0.009**	0.045
No	284/61.3	167/60.1	117/62.6		102/67.1	88/56.8	97/61.0		104/69.3	78/52.0	102/62.6		
Yes	179/38.7	111/39.9	68/36.8		50/32.9	67/43.2	62/39.0		46/30.7	72/48.0	61/37.4		
Preferred age Median (Range)	*n* = 179	*n* = 111	*n* = 68		*n* = 50	*n* = 67	*n* = 62		*n* = 46	*n* = 72	*n* = 61		
Mean ± SD	40 (27–50)	40 (30–50)	40 (27–50)		40 (30–50)	40 (27–50)	43.5 (30–50)		40 (27–50)	40 (30–50)	45 (30–50)		
	41.5 ± 5.5	41.6 ± 5.2	41.2 ± 6.0		39.1 ± 5.4	41.6 ± 5.3	43.3 ± 5.1		39.9 ± 5.8	40.9 ± 5.9	43.4 ± 4.2		
… a desired work experience in years (preferred work experience in years: Mean ± SD)													
	*n* = 249	*n* = 154	*n* = 95	0.637	*n* = 74	*n* = 91	*n* = 84	0.544	*n* = 72	*n* = 77	*n* = 100	0.149	0.061
Mean ± SD	9.0 ± 5.8	9.0 ± 6.2	8.9 ± 5.1		7.5 ± 4.2	9.6 ± 7.2	9.6 ± 5.1		6.0 ± 3.7	11.6 ± 6.4	9.1 ± 5.6		
**How important is …**													
… a welcoming reception in the dental office to you?													
	*n* = 465	*n* = 278	*n* = 187	**<0.001**	*n* = 151	*n* = 155	*n* = 159	0.588	*n* = 152	*n* = 149	*n* = 164	ANOVA **0.001** Bonferoni B * L < **0.001** B * M **0.003** L * M **0.002**	0.186
Very unimportant	0/0	0/0	0/0		0/0	0/0	0/0		0/0	0/0	0/0		
Unimportant	1/0.3	1/0.4	0/0		0/0	1/0.6	0/0		1/0.7	0/0	0/0		
Partly/partly	43/9.2	11/4.0	32/17.1		17/11.3	11/7.1	15/9.4		2/1.3	33/22.1	8/4.9		
Important	288/61.9	169/60.8	119/63.6		92/60.9	105/67.7	91/57.2		85/55.9	86/57.7	117/71.3		
Very important	133/28.6	97/34.8	36/19.3		42/27.8	38/24.5	53/33.3		64/42.1	30/20.1	39/23.8		
… it to you that the dental office staff and the dentist interact with each other in a friendly manner?													
	*n* = 466	*n* = 279	*n* = 187	**0.002**	*n* = 152	*n* = 155	*n* = 159	0.824	*n* = 152	*n* = 150	*n* = 164	ANOVA **<0.001** Bonferoni B * L **< 0.001** L * M **< 0.001**	0.2
Very unimportant	0/0	0/0	0/0		0/0	0/0	0/0		0/0	0/0	0/0		
Unimportant	15/3.2	8/2.9	7/3.7		5/3.3	4/2.6	6/3.8		9/5.9	3/2.0	3/1.8		
Partly/partly	81/17.4	30/10.8	51/27.3		26/17.1	27/17.4	28/17.6		13/8.6	66/44–0	2/1.2		
Important	268/57.5	174/62.4	94/50.3		83/54.6	98/63.2	87/54.7		84/55.3	67/44.7	117/71.3		
Very important	102/21.9	67/24.0	35/18.7		38/25.0	26/16.8	38/23.9		46/30.3	14/9.3	42/25.6		

## Data Availability

The data presented in this study are available from the corresponding author upon request. The data are not publicly available due to ethical reasons.
